# Smart Adsorption-Based Nanocatalysts for Active Food Packaging: A Critical Look at the Gap Between Concept and Application

**DOI:** 10.3390/nano16160980

**Published:** 2026-08-10

**Authors:** Amir Khojastehnezhad, Maziar Jafari, Fatemeh S. Mohseni-Shahri, Farid Moeinpour, Mohamed Siaj

**Affiliations:** 1 Department of Chemical Engineering and Biotechnological Engineering, Université de Sherbrooke, 2500 Boulevard de l’Université, Sherbrooke, QC J1K 2R1, Canada; akhojastehnezhad@yahoo.com; 2 Department of Chemistry, University of Quebec at Montreal, Montreal, QC H3C 3P8, Canada; jafari.maziar@courrier.uqam.ca; 3 Department of Chemistry, Islamic Azad University, Bandar Abbas, Iran; fs.mohseni@iau.ir

**Keywords:** smart adsorption-based nanocatalysts (SABNs), active food packaging, ethylene scavenging, stimuli-responsive materials, evidence grading, nanoparticle migration, food safety

## Abstract

Conventional food packaging cannot actively regulate spoilage-related molecules such as ethylene and volatile organic compounds that accumulate inside sealed packages. Smart adsorption based nanocatalysts (SABNs) integrate adsorptive scaffolds, catalytic centers, and stimuli responsive triggers to progressively remove these spoilage markers. This review establishes a unified three pillar framework and critically examines how adsorption, catalytic degradation, and regeneration cycles are proposed to function under food-relevant conditions. Across major food categories, reported photocatalytic systems achieve ethylene removal efficiencies of 50% to 90% and extend shelf life by 1 to 5 days under controlled light and temperature. However, performance declines sharply under the dark, humid, and refrigerated conditions typical of real supply chains. A systematic evidence level grading of twelve representative SABN systems reveals that the majority cluster at levels L^3^ and L^4^, while none has yet reached level L^5^, which requires both standardized migration testing and sensory evaluation. Key barriers, including nanoparticle migration, fragmented regulation, scalability, and life-cycle impacts, are assessed. By introducing explicit inclusion/exclusion criteria and a six-level evidence grading framework, this review maps critical gaps in migration data and cold-chain validation and outlines a staged roadmap toward regulation-ready active packaging technologies.

## 1. Introduction

Despite improvements in refrigeration and logistics, global food loss remains unacceptably high [1]. The environmental burden of conventional plastic packaging adds urgency to the search for functional alternatives. Globally, the packaging sector accounts for approximately 36% of all plastic produced, and food packaging represents the largest single component of this stream. An estimated 40% of plastic packaging waste is landfilled, while roughly 32% leaks into the environment, with at least 8 million tons entering the oceans each year [2]. These figures underline the fact that passive barrier films not only fail to extend shelf life sufficiently but also contribute to a persistent waste problem. Materials that actively manage the package headspace could, in principle, reduce both food loss and the environmental footprint of the packaging itself. Traditional plastic films can limit oxygen and moisture transfer; however, they do little to neutralize agents produced inside, a ripening banana still emits ethylene; cheese still accumulates off-flavor volatiles; grains still develop mycotoxins [3,4]. Over the last decade, researchers have begun to incorporate nanomaterials into packaging to address these internal threats [5,6]. However, most efforts have focused either on passive adsorption (e.g., ethylene scavengers) or on stand-alone catalysis (e.g., photocatalytic TiO_2_) [7]. In practice, such single-function approaches often lack durability or selectivity. In Li et al. (2020), it was observed that combining TiO_2_ nanoparticles with a tailored ZIF-8 scaffold can simultaneously enhance the adsorption and degradation efficiency [8]. Additionally, Faraldos and Bahamonde (2017) demonstrated that adding graphene oxide to TiO_2_ to form a GO–TiO_2_ composite significantly enhances catalytic performance under visible light, improving the charge separation, surface area, and adsorption capacity [9].

In our view, smart adsorption-based nano catalysts (SABNs), which merge high-surface-area adsorbents with catalytic centers and include a stimuli-responsive switch, offer a more holistic strategy [10,11]. By adsorbing target molecules (ethylene, VOCs, mycotoxins) and then catalytically degrading them (via photocatalysis, nanozyme pathways, or other mechanisms), SABNs can continuously cleanse the package interior [12,13,14]. For instance, Du et al. (2023) showed that when a SABN is overcoated with a pH-responsive nanofilm, it changes activity upon pH buildup and self-regenerates after contaminant adsorption [12]. The smart component (pH sensitivity, light activation, humidity triggers) allows for regeneration or tuning of activity according to evolving headspace conditions [12]. While several recent reviews have thoroughly covered photocatalytic films, MOF-based adsorbents, and intelligent freshness indicators individually, none has examined the deliberate integration of all three functions, adsorption, catalytic degradation, and stimuli-responsive regeneration, as a unified design problem. The present review addresses this gap by introducing the SABN framework and by critically appraising the evidence through original inclusion/exclusion criteria and an evidence-quality grading system. A fuller justification of this research gap is provided in Section 2.5.

Against this background, the present review has four specific aims: (i) to introduce a unified three-pillar framework for SABNs, together with explicit inclusion and exclusion criteria that define this emerging material class; (ii) to critically evaluate the mechanistic and practical evidence for SABNs under food packaging conditions; (iii) to apply an evidence-level grading system that systematically identifies translational gaps, particularly in migration testing and cold-chain performance; and (iv) to outline a staged roadmap for moving the most promising SABN candidates toward regulatory and commercial readiness. The remainder of the manuscript is structured as follows. Section 2 defines SABNs and establishes the conceptual framework. Section 3 discusses design principles, while Section 4 examines adsorption, degradation, and regeneration mechanisms. Section 5 critically evaluates reported applications across major food categories. Section 6 addresses commercialization barriers, safety, regulation, and life-cycle considerations. Finally, Section 7 synthesizes the main findings and proposes a forward-looking research roadmap.

## 2. Background and Scope

### 2.1. Why Conventional Packaging Falls Short

It is estimated that roughly one-third of all food produced worldwide never reaches a consumer [1,15]. Even under refrigeration, endogenous gases (notably ethylene), microbial metabolites (amines, sulfides), and chemically stable toxins (aflatoxins, fumonisins) continue to accumulate, ultimately degrading the quality [3,16]. Passive barrier films—made from polyethylene, polypropylene, or polyethylene terephthalate—can postpone water loss or oxygen ingress, but they do not actively dismantle spoilage species [17]. Thus, materials that both capture and neutralize internal threats are urgently needed to reduce waste and preserve food safety [18].

### 2.2. From Passive to Active and Intelligent Packaging

The term “active packaging” has come to encompass any film or insert that intentionally interacts with the headspace or surface of the food. Examples include oxygen or moisture scavengers, antimicrobial agents (e.g., silver nanoparticles), and ethylene absorbers (e.g., potassium permanganate sachets) [11,12]. “Intelligent packaging,” by contrast, integrates sensors or indicators such as colorimetric freshness strips that report on real-time quality [19]. Although these paradigms sometimes overlap, very few systems have united adsorption, catalysis, and sensing into a single entity. That gap is where SABNs belong [18]. It is important to note that sensing and reporting of spoilage, the core functions of intelligent packaging, are distinct from the adaptive catalytic modulation that defines the “smart” function in SABNs.

### 2.3. The Promise of Nanotechnology

Nanostructured materials offer unique advantages for active food packaging because their high surface-to-volume ratio and tunable surface chemistry enable efficient adsorption of spoilage-related molecules such as ethylene, VOCs, and microbial metabolites [10,12,20,21,22]. Different classes of nanomaterials can provide complementary functions; for example, some facilitate light-driven oxidative reactions [23,24], while others offer abundant adsorption sites or exceptionally high porosity. However, when deployed singly, each class exhibits limitations: pure adsorbents eventually saturate and lose efficacy, whereas stand-alone photocatalysts may generate reactive oxygen species that inadvertently attack the food [25,26]. Therefore, integrating adsorption with catalytic degradation while adding a smart trigger overcomes these shortcomings, creating systems that both capture and convert spoilage molecules in situ [12]. This synergy forms the conceptual foundation of SABNs. These trends are consistent with our previous findings on nanostructured adsorbents and catalytic hybrids for food-relevant environments [27,28].

### 2.4. Defining Smart Adsorption-Based Nanocatalysts (SABNs)

In this review, the term smart adsorption-based nanocatalysts (SABNs) is reserved for materials that simultaneously and intentionally integrate three functional components within a single platform: (i) an adsorptive scaffold, (ii) a catalytic center, and (iii) a stimuli-responsive mechanism. A stimuli-responsive mechanism enables the SABN to modulate its activity in a reversible switch-like manner when triggered by environmental cues (e.g., light, pH, humidity). This capacity for on-demand activation and deactivation, rather than mere passive sensitivity, is what distinguishes the ‘smart’ function from conventional stimuli-responsive materials. The three pillars must operate synergistically: adsorption enriches target molecules at the catalytic site, catalysis transforms them into benign products, and the stimuli-responsive element modulates activity or enables regeneration under dynamically changing headspace conditions. A material that satisfies only one or two of these criteria is not classified as an SABN in the present framework. It should be noted that antimicrobial agents that act via ion release (e.g., Ag^+^) or stoichiometric oxidation do not qualify as catalytic centers, as they are consumed during use and do not exhibit sustained turnover. A concrete example helps to illustrate how these three pillars interact in practice. Hong et al. (2025) developed a composite film in which TiO_2_ nanoparticles were embedded within a cerium-based metal–organic framework (Ce-MOF) [29]. In this system, the microporous MOF scaffold served as the adsorptive component, selectively concentrating ethylene molecules from the headspace and bringing them into close proximity with the TiO_2_ catalytic sites. The TiO_2_ component, upon exposure to UV light, generated reactive oxygen species that oxidized the adsorbed ethylene. The Ce-MOF scaffold was not merely a passive support; its redox-active Ce^3+^/Ce^4+^ centers contributed to catalytic turnover and, importantly, provided a humidity-responsive characteristic that modulated activity under moist conditions. What makes this system an SABN rather than a simple catalyst-adsorbent mixture is that the three functions were designed to work together: the scaffold captured and concentrated the target, the catalytic sites transformed it, and the responsive element adapted the system to changing environmental conditions. To resolve this conceptual ambiguity and provide a consistent analytical lens throughout the review, we propose the following operational inclusion and exclusion criteria (Table 1). This classification framework is a conceptual contribution of the present work, logically derived from the three-pillar definition introduced above. It is designed to distinguish true SABNs from the broader landscape of nanocomposite materials and should not be taken as a universal taxonomy proposed elsewhere.

**Figure 1:**
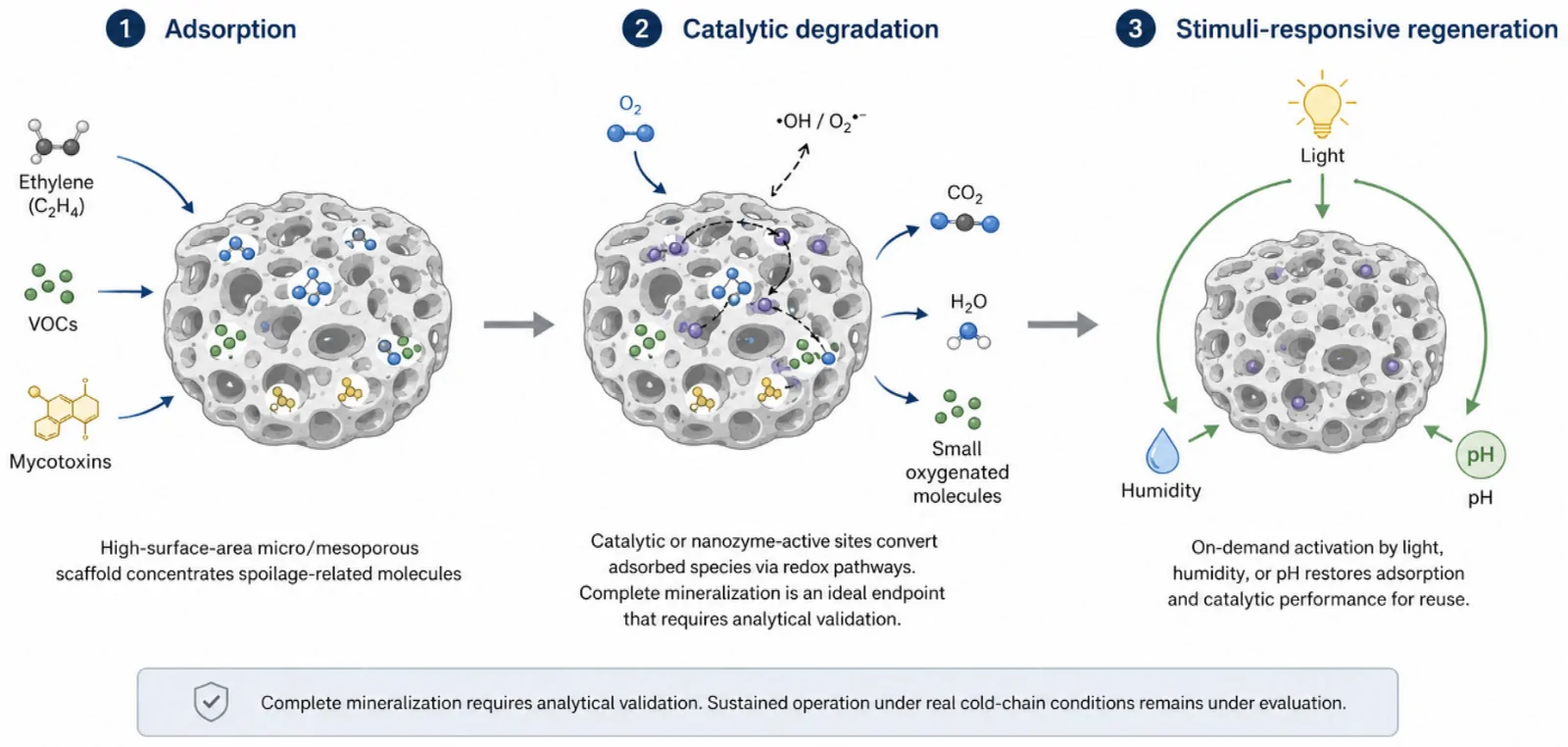
Conceptual framework of SABNs in active food packaging, integrating adsorption, catalytic conversion, and stimuli-responsive regeneration within a single platform. This three-pillar architecture is designed to enhance selectivity, delay saturation, and support reusable performance in response to external triggers. Complete mineralization and sustained operation under real cold-chain conditions remain areas for further validation.

This classification framework serves a dual purpose. First, it ensures that the term SABN is applied consistently throughout the review. Second, it allows readers to distinguish genuine SABNs from the broader landscape of nanocomposite active packaging materials, many of which are functional but do not embody the three-pillar synergistic design that defines the SABN concept. Figure 1 schematically illustrates the core components and operating principles of SABNs. In subsequent sections, each reviewed system is mapped against these criteria. These criteria make it possible to distinguish SABNs from related but distinct packaging categories in a straightforward way. A conventional active film that contains a photocatalyst but lacks a deliberately designed adsorptive scaffold and a stimuli-responsive trigger does not qualify as an SABN. Intelligent indicators, which sense and report spoilage but do not catalytically degrade the target molecules, also fall outside the definition. Likewise, ordinary nanocomposite films that improve barrier or mechanical properties without the synergy of adsorption, catalysis, and responsiveness are not SABNs. The framework thus draws a clear line around the much smaller set of materials that genuinely attempt to integrate all three functions. With a clear definition and explicit classification criteria now in place, the next logical question is why a dedicated review on SABNs is needed at all, a justification we develop in detail in Section 2.5.

### 2.5. Rationale for a Dedicated Review

The need for a unified treatment of SABNs becomes apparent when surveying the existing review literature. In recent years, several high-quality reviews have substantially advanced our understanding of individual functional modules relevant to active packaging. For instance, Hua et al. (2024) provided a comprehensive overview of photocatalytic ethylene scavenging, focusing almost exclusively on light-driven catalytic degradation [30]. In parallel, Yang et al. (2024) surveyed the rapidly expanding use of metal–organic frameworks (MOFs) in food packaging, a body of work that emphasizes selective adsorption and antimicrobial properties [10]. Du et al. (2023) examined smart active packaging systems with an emphasis on stimuli-responsive triggers and regeneration mechanisms [12]. Together, these and other overviews [31,32] have thoroughly mapped the territories of adsorption, photocatalysis, and environmental responsiveness, but they have done so largely in isolation from one another.

While the synergy between adsorption and catalysis has been widely exploited in outstanding materials such as MOF composites and metal oxide hybrids, the explicit integration of a stimuli-responsive trigger, enabling on-demand regeneration or adaptive performance, remains rare in food packaging research. What is conspicuously absent, therefore, is a review that treats all three functions, adsorption, catalytic degradation, and stimuli-responsive regeneration as interdependent design requirements that must be engineered simultaneously and that critically evaluates how far such integrated systems have actually progressed. In a real food package, these functions do not operate independently: the performance of the catalyst depends on how efficiently the scaffold concentrates the target molecule, and the longevity of the system depends on whether the responsive trigger can regenerate the active sites. Reviewing any one pillar without the others provides an incomplete picture of the challenge. The present review therefore attempts to bridge this gap by adopting a deliberately integrated three-pillar perspective. Specifically, we aim to (i) trace how diverse nanomaterials have been combined to create hybrids capable of simultaneous adsorption and catalytic transformation; (ii) clarify the underlying mechanisms from adsorption isotherms to reactive-oxygen-species generation and highlight how structural and chemical design choices influence performance; (iii) present representative applications across different food categories, emphasizing realistic operating conditions; and (iv) identify key bottlenecks, including safety, scalability, cost, and regulatory constraints, while outlining a critical roadmap for future research. Having justified the need for an integrated perspective, we now move to the design principles that govern how the three SABN pillars can be engineered together (Section 3).

## 3. Design Principles for SABNs

Building a functional SABN is not simply a matter of mixing an adsorbent, a catalyst, and a responsive polymer into a single film. The three components must be treated as interdependent design variables from the outset. Table 2 offers a condensed summary of what each pillar contributes, the material families most commonly explored for that role, and the engineering trade-offs that must be managed. It serves as a quick reference for the more detailed discussion that follows.

What Table 2 cannot convey, however, is how strongly the performance of one pillar depends on the design of the other two. This interdependence is what makes SABN engineering genuinely challenging and what sets it apart from simply stacking functional layers. The adsorptive scaffolds explored for SABNs can be broadly grouped into four families, each with distinct advantages and limitations. Mesoporous silicas, such as SBA-15 and MCM-41, offer exceptionally high surface areas and tunable pore sizes, making them attractive for fundamental studies of adsorption selectivity [35]. Metal–organic frameworks (MOFs) have emerged as perhaps the most versatile scaffold family; their crystalline, modular structures allow pore size and surface chemistry to be tailored for specific target molecules, and several MOFs have shown high ethylene and VOC uptake under conditions relevant to food packaging [10,33]. Carbon-based scaffolds, including graphene oxide and activated carbon, combine a high surface area with good mechanical properties, and their surface chemistry can be modified to enhance affinity for particular spoilage markers [36]. Finally, biopolymer-based scaffolds derived from cellulose, chitosan, starch, or pullulan have attracted attention because they can be processed into flexible films and coatings, and their surface functionality can be tuned through simple chemical modification [37]. The choice of scaffold for a given SABN application must balance the adsorption capacity, selectivity under humid conditions, compatibility with the chosen catalytic center, and processability into a packaging format. The adsorptive scaffold, for instance, does more than provide surface area. Its pore-size distribution determines whether a target molecule such as ethylene can even reach the catalytic sites, while its surface chemistry governs how strongly water vapor competes for those same sites under the high-humidity conditions typical of fresh-produce packaging. A scaffold optimized for dry gas streams may perform poorly inside a refrigerated package. Likewise, the catalytic center must be chosen not only for its intrinsic activity but also for its compatibility with the scaffold: a photocatalyst that requires UV light is of little use if the scaffold or the polymer matrix blocks that light, or if the food is stored in darkness. The stimuli-responsive outer layer adds another layer of complexity; it should open or activate when needed and remain inert during the majority of the product’s shelf life so that it does not itself become a source of migrants. The following subsections unpack each pillar in turn, with an emphasis on the quantitative parameters and structure–property relationships that must be considered if SABNs are to move from laboratory demonstrations to packages that function reliably in real supply chains.

## 4. Mechanistic Considerations Relevant to SABNs in Food Packaging

**Figure 2:**
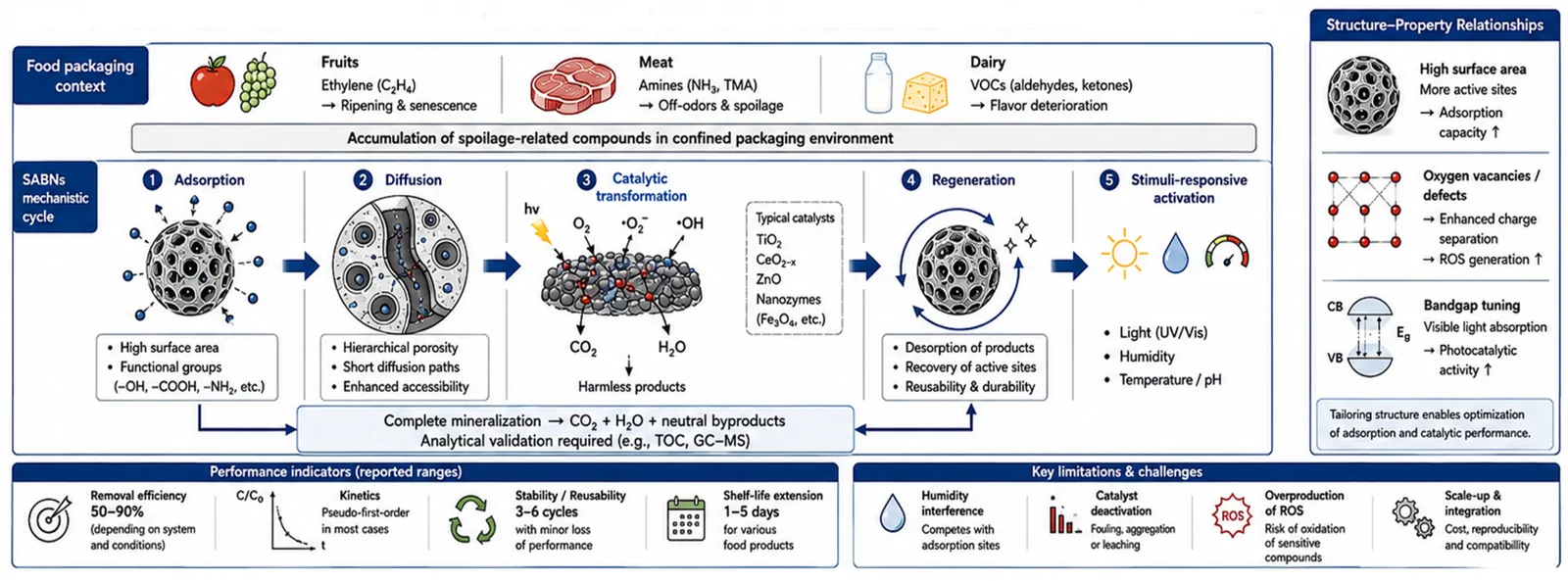
Coupled adsorption, diffusion, catalytic transformation, and regeneration enable effective ethylene and VOC removal under controlled laboratory conditions, but the performance may be reduced under the low-light, low-temperature, and high-humidity conditions common in real food supply chains. The schematic summarizes the principal mechanistic steps and the structure–property–performance relationships that govern SABN function.

SABNs operate through a coupled adsorption–diffusion–catalysis–regeneration cycle that enables continuous control of spoilage-related compounds within confined food packaging environments. As schematically illustrated in Figure 2, this integrated mechanism governs the capture, transformation, and removal of target molecules such as ethylene, volatile organic compounds (VOCs), and amines. The process is initiated by the selective adsorption of gaseous molecules onto high-surface-area nanostructures. This step is strongly influenced by structural parameters including the porosity, surface functional groups (e.g., –OH, –COOH, –NH_2_), and accessible active sites, as well as environmental factors such as humidity and temperature. High surface area and tailored surface chemistry enhance the adsorption capacity, but competitive adsorption with water molecules under high humidity can reduce the efficiency [36,38,39,40,41,42]. Following adsorption, molecular diffusion through hierarchical pore networks governs the transport of adsorbed species toward catalytically active sites. The diffusion efficiency depends on the pore size distribution, connectivity, and potential pore blockage caused by moisture accumulation or by-products. In food packaging environments, where the humidity is often elevated, diffusion limitations can significantly reduce the overall reaction rates [38,39]. Once molecules reach active sites, catalytic transformation occurs, typically via oxidation pathways. In many SABN systems, this process is mediated by the generation of reactive oxygen species (ROS), such as hydroxyl radicals (•OH) and superoxide radicals (O_2_^−^), which convert spoilage-related compounds into benign products such as CO_2_ and H_2_O [43,44,45,46]. The efficiency of this step depends on the catalytic material (e.g., TiO_2_, CeO_2−x_, ZnO, or nanozyme-based systems), light availability, and charge separation efficiency. For practical food packaging applications, catalysts must operate under low-intensity or intermittent light conditions and avoid the formation of harmful intermediates. Among the various catalytic materials employed in SABNs, TiO_2_ remains the most extensively studied photocatalyst for food packaging applications. Hua et al. (2024) reviewed photocatalytic ethylene scavenging and confirmed that TiO_2_ is favored for its stability and low cost, though its wide band gap limits activity to the UV region [30]. Faraldos and Bahamonde (2017) showed that combining TiO_2_ with graphene oxide can enhance visible-light response and improve charge separation, thereby increasing the ethylene degradation rates [9]. In a practical packaging context, Kaewklin et al. (2018) demonstrated that chitosan films loaded with TiO_2_ nanoparticles delayed tomato ripening by approximately six days compared to LDPE, although the study also noted that the photocatalytic benefit depended strongly on adequate light exposure [43]. For volatile organic compounds, complete mineralization to CO_2_ and H_2_O is the ideal endpoint, but in practice, oxidation frequently stops at intermediate aldehydes or organic acids, especially under the low photon flux and low oxygen concentrations typical of food packages. A distinguishing feature of SABNs is their stimuli-responsive activation, whereby environmental triggers such as light, humidity, or temperature dynamically regulate catalytic activity [43,47,48,49]. This responsiveness enables the adaptive control of reaction rates in response to changing storage conditions. However, the precise tuning of response thresholds remains challenging, and overproduction of reactive species may pose risks to food quality or packaging integrity. The final stage involves the partial regeneration of active sites through the desorption of reaction products and recovery of catalytic functionality. While many systems demonstrate reusability over multiple cycles, long-term stability can be compromised by catalyst deactivation mechanisms such as aggregation, fouling, or oxidation [41,45]. The overall efficiency of the SABN cycle is strongly governed by structure–property relationships. A high surface area enhances the adsorption capacity, oxygen vacancies and defect sites promote ROS generation, and bandgap tuning enables activation under visible light, an essential requirement for real-world packaging conditions [47,48]. Despite promising mechanistic performance under laboratory conditions, significant discrepancies remain between controlled experiments and real cold-chain environments. Most studies are conducted under idealized illumination, controlled humidity, and static temperatures, whereas actual food storage involves low-light conditions, fluctuating temperatures, and high moisture levels that can suppress catalytic activity or hinder diffusion [44,50]. It is worth noting that complete mineralization to CO_2_ and H_2_O is not always achieved under the conditions typical of food packaging. In confined, low-light, and high-humidity environments, the supply of reactive oxygen species is often insufficient to drive oxidation all the way to the final products. Several studies have reported that the photocatalytic degradation of ethylene and VOCs can produce partially oxidized intermediates such as formaldehyde, acetaldehyde, acetic acid, or formic acid (Faraldos and Bahamonde, 2017 [9]; Hua et al., 2024 [30]). These intermediates may themselves affect food flavor or safety, and their accumulation in a sealed package is a concern that has received relatively little attention in the SABN literature. For dark storage conditions, non-photocatalytic systems based on nanozymes such as CeO_2−x_ or Fe_3_O_4_ are more appropriate, as they can generate ROS via surface redox reactions without requiring photon flux. The disappearance of a target molecule from the headspace should not, therefore, be automatically interpreted as proof of complete detoxification. Direct product analysis for example by gas chromatography–mass spectrometry or total organic carbon measurement is needed to confirm that the overall catalytic process is genuinely beneficial. Consequently, mechanistic insights derived from laboratory studies do not always translate directly to practical performance. Incorporating cold-chain-relevant testing protocols into mechanistic investigations is therefore essential to evaluate the real-world applicability of SABNs. As shown in Figure 2, the performance of these systems emerges from the dynamic coupling of adsorption, diffusion, catalytic transformation, and regeneration processes, all of which are modulated by structural design and environmental conditions.

## 5. Applications of SABNs in Food Packaging: Opportunities and Critical Challenges

SABNs have been explored across a wide range of food categories, where they are primarily valued for their ability to modulate headspace composition by removing ethylene, volatile organic compounds (VOCs), and spoilage-related metabolites. Although numerous studies report meaningful improvements in shelf life and sensory attributes, the evidence remains uneven, and several methodological and practical limitations complicate translation to real supply-chain environments. Table 3 provides a quick orientation to the spoilage challenges, commonly studied SABN material types, and typical testing conditions associated with each major food category discussed in the following subsections.

### 5.1. Fruits and Vegetables

**Figure 3:**
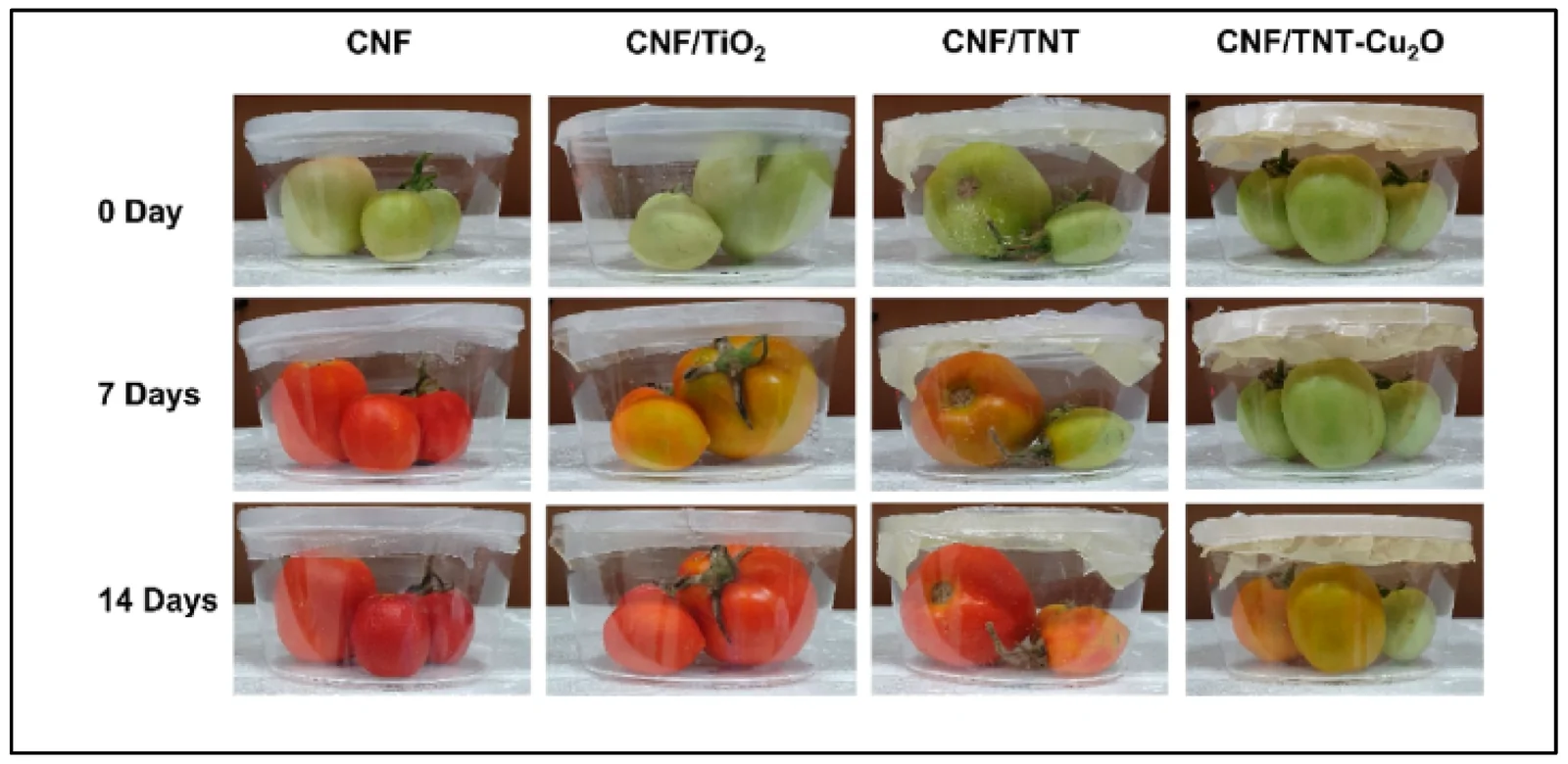
Delayed color change in tomatoes packaged with CNF/TNT-Cu_2_O films illustrates the potential of SABNs to slow ripening; however, visual appearance alone cannot be taken as direct proof of ethylene scavenging. Multiple factors, including film permeability, moisture retention, and antimicrobial effects, may contribute to the observed result. Reproduced with permission [51]. Copyright 2022 American Chemical Society.

In fresh-produce systems, SABNs have been most extensively studied for ethylene management and the removal of aldehydes associated with early senescence [49,51,52,53,54]. Packaging trials frequently report delayed ripening, improved firmness, reduced weight loss, and more stable color development. A representative example is shown in Figure 3, where tomato fruits wrapped in CNF/TNT-Cu_2_O films remained visibly greener after 14 days of storage at 25 °C [51]. While this observation is consistent with delayed ripening, it should not be taken as direct proof of ethylene-scavenging by the SABN alone. Tomato ripening is governed by multiple interacting factors including the respiration rate, moisture retention, film permeability, and potential antimicrobial effects of copper species that can influence the color development independently of ethylene removal. Representative systems such as TiO_2_@BMOF films (~90% ethylene removal, 5-day banana shelf-life extension) and the CNF/TNT-Cu_2_O film discussed above are detailed in Table 4. These examples highlight the promise of photocatalytic SABNs under controlled laboratory conditions; however, as critically examined in Section 5.5, such demonstrations are overwhelmingly confined to light-exposed small-scale settings that may not reflect commercial cold-chain realities.

### 5.2. Meat and Seafood

In protein-rich foods, SABNs have been applied to suppress microbial metabolites and reduce amine-based off-odors [55,56,57,58]. These findings are important, because odor control is a major driver of perceived freshness. For instance, active films have been shown to reduce amine-based off-odors in seafood model systems (see Table 4). However, the extrapolation of these findings to intact muscle foods under refrigerated, high-humidity, and dark storage conditions typical of real supply chains remains a significant uncertainty. The specific methodological limitations hampering translation are discussed in detail in Section 5.5.

### 5.3. Dairy and Bakery

**Figure 4:**
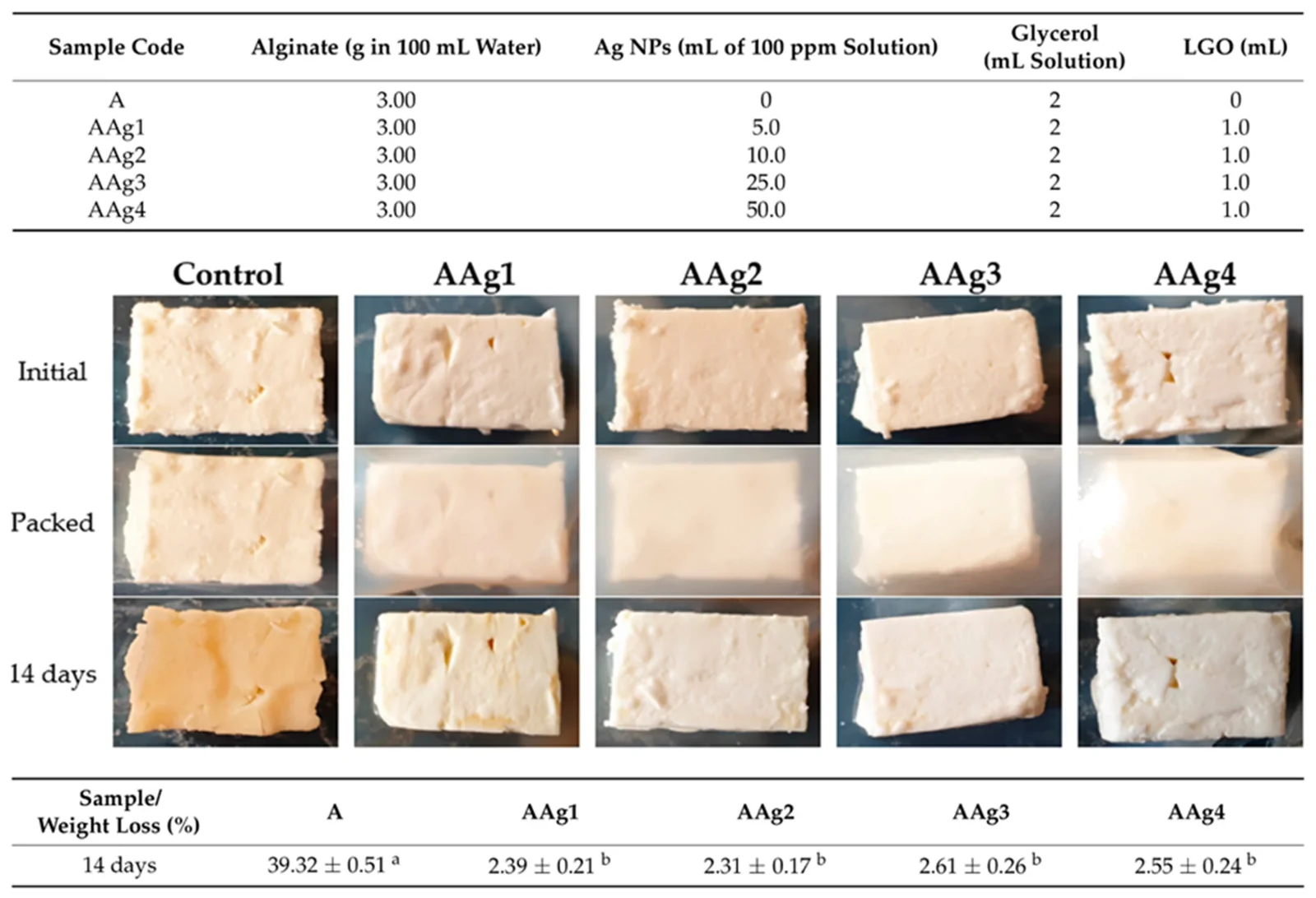
Images of curd cheese comparing color and texture after storage in bare alginate and alginate–AgNPs–lemongrass essential oil (AAg) composite films at varying AgNPs concentrations. The figure illustrates the improved white color retention after 14 days of refrigerated storage in 75% relative humidity, including the soft and moist textural appearance. The resulting samples wrapped with the composites exhibited lesser mass loss (drying) and pH retention following the 2-week period. Reprint from Ref. [70]. Different superscript letters in the same column indicate statistically significant differences between films (*p* < 0.05).

Active films have been shown to reduce VOCs linked to rancidity and staling, and some case studies report improved sensory outcomes in cheese and bread [21,59,60,61,62,63,64,65,66,67,68,69]. Figure 4 (Motelica et al.) is a useful demonstration of visual and textural quality differences in cheese under active packaging [70]. Studies such as those by Motelica et al. (2021) on alginate-AgNPs-lemongrass essential oil (AAg) films for cheese preservation have shown promising visual, textural, mass-retention and beneficial pH outcomes (see Figure 4 and Table 4) [70]. Yet, the translation to commercial dairy and bakery products is hindered by impending questions regarding whether the extent of AgNPs leaching potentially leads to toxicity, or if off-flavor effects, stability of active functions in dark refrigeration, and potential matrix interactions are influential parameters. These challenges are not unique to this category and are systematically analyzed in Section 5.5.

### 5.4. Multilayer Formats, Sachets and Indicators

Multilayer laminates, active sachets, and hybrid indicator systems offer attractive modular integration pathways for SABNs into existing packaging lines [62,71,72,73,74,75,76,77,78,79]. For a comparative performance overview of these and other systems, see Table 4. However, these formats introduce acute regulatory and end-of-life complexities. The migration of active agents across multilayer interfaces and the recyclability of hybrid films remain poorly characterized. Natural indicators (e.g., anthocyanin-based labels) are promising for consumer communication but are vulnerable to false positives/negatives under fluctuating humidity and light; without robust validation, these indicators risk eroding consumer trust rather than enhancing it.

### 5.5. Comparative Performance of SABNs

To enable a more systematic evaluation of reported systems, a comparative summary of representative SABNs is presented in Table 4. The comparison focuses on key performance indicators, including removal efficiency, target compounds, operational conditions, and reported shelf-life extension. While some studies report quantitative removal efficiencies, others primarily demonstrate functional performance (e.g., shelf-life extension or quality preservation), highlighting the lack of standardized evaluation metrics across SABN systems. This variability, and its implications for translational credibility, are critically examined in Section 5.6 and throughout Section 6.

**Table 4:** Comparative performance of SABNs under diverse experimental conditions in food packaging applications.

SABN System	Core Components (Scaffold/Catalyst /Stimuli)	Target Molecule(s)	Test Condition	Key Performance	Food Application and Effect	Migration Tested?	Refs.
TiO_2_@BMOF chitosan/gelatin film ^L3^	Scaffold: Biopolymer; Catalytic: TiO_2_ on Bi-MOF; Stimuli: Light	Ethylene (C_2_H_4_)	20 °C, ~55% RH, Visible light (1000 lux), Packaged banana headspace	~90% ethylene removal in 24 h	Banana; 5-day shelf-life extension, reduced browning and firmness loss	N.R.	[67]
Ce-MOF/TiO_2_ composite film ^L3^	Scaffold: Ce-MOF; Catalytic: TiO_2_; Stimuli: UV light	Ethylene (C_2_H_4_)	25 °C, Dark/UV (365 nm, 8 W), Sealed container (100 ppm ethylene)	Complete removal of 100 ppm C_2_H_4_ in 6 h under UV	Avocado; Delayed ripening, maintained firmness	N.R.	[29]
Zeolite–TiO_2_ coated paper ^L3^	Scaffold: Zeolite; Catalytic: TiO_2_; Stimuli: Light-assisted	Ethylene (C_2_H_4_)	Room temp, Fluorescent light (2000 lux), Tomato headspace	~3× increase in ethylene adsorption capacity vs. pristine paper	Tomato; ~3× shelf-life extension compared to uncoated paper	N.R.	[54]
PLA/TiO_2_ nanocomposite film ^L3^	Scaffold: PLA matrix; Catalytic: TiO_2_ NPs; Stimuli: Ambient light	Ethylene (C_2_H_4_)	23 ± 2 °C, Ambient light, Packaged banana	Measurable ethylene reduction (GC analysis); preserved banana quality for 10 days	Banana; Maintained visual quality, delayed ripening for 10 days	N.R.	[80]
PAN@TiO_2_ electro spun nanofibers ^L3^	Scaffold: PAN nanofibers; Catalytic: TiO_2_; Stimuli: UV/light	Ethylene (C_2_H_4_)	Room temp, UV light (365 nm), Sealed batch reactor	Efficient photocatalytic degradation (approx. 80% in 120 min under UV)	Model fruit (potential packaging); High preservation potential demonstrated	N.R.	[81]
CNF/TNT–CuO film ^L3^	Scaffold: Cellulose nanofiber; Catalytic: TiO_2_ nanotubes/CuO; Stimuli: Light	Ethylene (C_2_H_4_)	25 °C, Room light, Packaged tomato	Significant delay in tomato color change (remained green after 14 days)	Tomato; Ripening significantly delayed vs. neat CNF film (visual)	N.R.	[51]
Chitosan/TiO_2_ nanocomposite film ^L3^	Scaffold: Chitosan; Catalytic: TiO_2_; Stimuli: Light	Ethylene, VOCs	20 °C, Fluorescent light, Packaged tomato	Not quantified directly; significantly delayed ripening and reduced weight loss	Tomato; Shelf life prolonged by ~6 days vs. LDPE	N.R.	[43]
Zein–TiO_2_ electro spun nanofibers ^L3^	Scaffold: Zein protein; Catalytic: TiO_2_; Stimuli: Light	Ethylene (C_2_H_4_)	25 °C, UV-A light (1.5 mW/cm^2^), Cherry tomato pack	Photocatalytic degradation of ethylene (approx. 65% in continuous flow)	Cherry tomato; Delayed firmness loss and color change for 10 days	N.R.	[75]
Ag/graphene/TiO_2_-PLA composite film ^L4^	Scaffold: PLA; Catalytic: TiO_2_, Ag, graphene; Stimuli: Light	VOCs, microbial metabolites (in cheese)	4 ± 1 °C, Dark (refrigerated), Curd cheese contact	Organoleptic quality maintained; reduced whey leakage and mottling vs. PP	Cheese; Better appearance and less spoilage after 17 days	N.R. (Ag release not specifically studied for migration limits)	[60]
Alginate–AgNPs–lemongrass essential oil ^L3^	Scaffold: Alginate; Catalytic: AgNPs, LGO; Stimuli: Hyperpolarization, pH	VOCs, Microbial spoilage (in cheese)	4 ± 1 °C, 75% RH (refrigerated)	Significant reduction in mass loss; original pH, texture, and color retention	Cheese; Better appearance compared to control and improved shelf life up to 14 days	N.R. (AgNPs release not specifically studied for migration limits)	[70]
TiO_2_/cinnamon essential oil bio-nanocomposite film ^L4^	Scaffold: Biopolymer; Catalytic: TiO_2_; Stimuli: Light?	VOCs, microbial spoilage	Refrigerated (4 °C), Dark, Sliced cheese contact	Maintained sensory and microbial quality; lower peroxide value	Cheese; Shelf life extended by ~4 days vs. control film	N.R.	[65]
pH-responsive nanozyme pad (CeO_2_@nanocellulose) ^i L4^	Scaffold: Nanocellulose; Catalytic: CeO_2_ nanozymes; Stimuli: pH (spoilage-induced)	Biogenic amines (e.g., trimethylamine)	4 °C, Dark, Fresh fish fillet (intact tissue)	Reduced TVB-N by ~40% after 7 days; colorimetric signal change	Fish fillet; Freshness (sensory) extended by 2–3 days under cold storage	N.R. (Particle release in moist contact not tested)	Hypothetical composite based on [57,82] ^ii^
Commercial KMnO_4_-based sachet (benchmark) ^L3^	Scaffold: Porous sachet; Catalytic: KMnO_4_ (oxidation); Stimuli: None (passive)	Ethylene (C_2_H_4_)	Ambient, Dark, Fruit box	Oxidation capacity: ~20 mL C_2_H_4_/g sachet; no regeneration	Wide range of fruits; Proven shelf-life extension (days)	N.R. (Not nanosized)	(commercial)

Abbreviations: N.R. = not reported; RH = relative humidity; GC = gas chromatography; TVB-N = total volatile basic nitrogen; CNF = cellulose nanofiber; TNT = TiO_2_ nanotubes; PP = polypropylene; LDPE = low-density polyethylene; LGO = lemongrass essential oil; PLA = polylactic acid. Footnotes: ^i^ This row is a representative SABN concept integrating CeO_2_ nanozymes with a pH-responsive scaffold. Specific quantitative ethylene/VOC removal data under real food conditions are scarce; the values presented are exemplary of typical nanozyme/peroxidase activity from the cited mechanistic studies. ^ii^ While direct food packaging data for this specific composite are limited, the catalytic and responsive principles are well-established in [82] (CeO_2_/TiO_2_ nanozymes) and [57] (CeO_2_–chitosan films). Evidence levels (superscript after system name): ^L1^ = Catalyst-only reactor test (no food matrix); ^L2^ = Model headspace simulation; ^L3^ = Real food, ambient light/room temperature; ^L4^ = Real food, dark/refrigerated storage; ^L5^ = L^4^ + standardized migration assay + sensory panel; ^L6^ = Pilot-scale fabrication + techno-economic or LCA analysis. A few further remarks on the level assignments may be helpful. The boundary between L^3^ and L^4^ is defined by storage environment: an L^3^ study uses real food under ambient light and room temperature, whereas an L^4^ study places the packaged food in darkness at refrigeration temperature and, ideally, at elevated humidity. The step from L^4^ to L^5^ is more demanding. To reach L^5^, a study must include both a standardized migration assay (preferably using recognized food simulants and, where possible, the actual food matrix) and a sensory evaluation conducted by a trained panel under controlled conditions. Studies that report only informal visual observation or that measure migration with a single simulant under unrealistic contact conditions were not classified as L^5^. Finally, L^6^ adds the requirement of pilot-scale fabrication together with a transparent cost analysis or life-cycle assessment. No study in the current literature meets these combined criteria. We hope this clarification makes the grading system more transparent and reproducible. Systems were selected to represent the diversity of adsorptive scaffolds, catalytic centers, and stimuli-responsive mechanisms reported in the food packaging literature. Selection prioritized the most cited and most application-relevant studies; no system was excluded on the basis of its evidence level.

### 5.6. Evidence-Quality Assessment Across SABN Application Studies

A closer look at Table 4 reveals more than just what each system achieved; it also tells us how much weight those achievements can carry. To make this judgement easier, we labelled each entry with a simple evidence-level tag (L^1^ to L^6^), the criteria for which are explained in the footnote of the table. In short, the scale runs from basic catalyst-only reactor experiments (L^1^) all the way to pilot-scale studies that include standardized migration testing, sensory panels, and a credible cost analysis (L^6^). Once the tags are in place, the pattern is hard to ignore. The majority of systems in Table 4 fall into the L^3^ category: real food was used, and the results often look encouraging, but the experiments were conducted under ambient light and room temperature conditions that have little in common with a refrigerated supply chain. A smaller number of studies reach L^4^, where the packaging was tested in the dark and at low temperature with actual food. What is completely absent, however, are systems that meet the requirements for L^5^ (which asks for both a proper migration assay and a trained sensory panel) or L^6^ (pilot-scale fabrication combined with techno-economic or life-cycle analysis). This distribution has a very practical consequence. A claim that a film “extends shelf life by three days” means something quite different when it comes from a L^3^ study than it would from a L^4^ or L^5^ study. Performance achieved under continuous light and moderate temperature cannot simply be assumed to hold in a dark, humid, refrigerated package, because the very triggers that drive catalysis light, heat, and oxygen availability are deliberately suppressed in the cold chain. The absence of L^5^ and L^6^ studies is not a criticism of the individual research groups; many of those papers were the first to demonstrate an exciting new material. It is, however, a sobering reflection of where the field as a whole currently stands. Looked at through this lens, Table 4 turns into a kind of priority list. The most urgent task facing the community is not to report yet another high-performing L^3^ catalyst under idealized light but to take the most promising L^3^ and L^4^ candidates and push them toward L^5^ by adding standardized migration tests and sensory evaluations under realistic storage and distribution conditions. Until that shift begins, the translational gap that this review has repeatedly pointed out will remain, and SABNs will stay in the laboratory rather than moving into the supermarket.

## 6. Commercialization, Scalability, and Life-Cycle Assessment

Despite significant advances in the development of SABNs, their transition from laboratory-scale concepts to commercially viable food packaging technologies remains limited. This gap is primarily driven by challenges related to the scalability, cost, safety, regulatory approval, and environmental sustainability. A critical and semi-quantitative assessment of these factors is essential to evaluate the realistic potential of SABNs. In the following subsections, we examine the technology readiness and scalability, safety and migration, regulatory and standardization issues, life-cycle implications, and socio-economic barriers and outline a roadmap for credible commercialization.

### 6.1. Technology Readiness, Scalability, and Cost

Most SABN systems are currently at low to intermediate technology readiness levels (TRL 2-4), where performance is demonstrated under controlled laboratory conditions but not validated in industrial environments. Scalability challenges arise from multi-step synthesis processes, the use of high-purity or doped precursors, and energy-intensive fabrication steps such as high-temperature calcination or solvothermal treatment. Photocatalytic nanomaterials such as doped TiO_2_ or hybrid composites often require tightly controlled synthesis conditions that are difficult to reproduce at scale, whereas simpler adsorption-based systems (e.g., permanganate sachets) are already commercially viable due to their low cost and established manufacturing routes. Most SABN syntheses remain confined to laboratory conditions, relying on high-temperature calcination or costly precursors that are incompatible with industrial economics. Although greener, low-temperature, or biogenic routes offer promising alternatives [66,83,84], their reproducibility and batch-to-batch consistency remain largely untested. Transitioning from batch processes to roll-to-roll or printing-based deposition [85,86] is frequently cited as a pathway to scalability, yet techno-economic analyses often omit critical cost drivers such as nanoparticle stabilization, quality control, and end-of-life handling. The performance data compiled in Table 4 reflect this nascent stage: while laboratory removal efficiencies of 80–90% are common, the conditions required to achieve them, for instance, continuous UV illumination in Zeolite–TiO_2_ or PAN@TiO_2_ systems are a direct indicator of the manufacturing and operational costs not yet accounted for in commercial projections. The economic viability of SABNs depends on the material cost, processing complexity, and integration into existing packaging systems. Estimated cost drivers include nanomaterial synthesis (moderate to high), functionalization and doping (high), and incorporation into films or coatings (moderate). Preliminary comparisons suggest that SABN-based systems may be 2–5 times more expensive than conventional active packaging solutions, depending on the material complexity. However, this cost may be justified in high-value applications where even 2–3 days of shelf-life extension leads to a significant reduction in food waste and economic loss. 

Beyond the technical readiness of the materials themselves, several commercialization barriers must be acknowledged. Production costs for SABNs are driven by the price of high-purity precursors, the energy demand of calcination or solvothermal steps, and the solvents consumed during multi-step synthesis. When these upstream costs are combined with the expenses of functionalization, doping, and quality control, preliminary estimates place SABN-based systems at two to five times the cost of conventional active packaging. Scalability is constrained by the fact that most SABN syntheses reported to date are batch processes that have not been demonstrated at pilot scale. Translating a laboratory preparation into a continuous roll-to-roll or reactive extrusion workflow without losing nanoparticle dispersion, catalytic activity, or film uniformity is a non-trivial engineering challenge. Processing constraints further complicate the picture. Many catalytic nanoparticles require high-temperature calcination that is incompatible with heat-sensitive biopolymer matrices, and nanoparticle agglomeration during film casting or extrusion can reduce both the mechanical integrity and active surface area. Market adoption adds a final layer of difficulty. Incumbent technologies such as potassium permanganate sachets and modified-atmosphere packaging are inexpensive, well understood, and already integrated into supply chains. Convincing packaging converters and retailers to switch to a more complex, more expensive, and less familiar SABN solution will require not only proven technical superiority but also a clear return on investment, which has not yet been demonstrated. Table 5 provides a high-level comparison of conventional petroleum-based films with SABN systems based on both conventional and bio-based polymer matrices.

A major limitation is the lack of comprehensive techno-economic analyses in the literature, meaning that projected cost–benefit ratios and return on investment remain uncertain. Cost-reduction models for sachets or antimicrobial patches [87,88] rarely incorporate regulatory testing or recycling burdens, meaning that projected savings may be overly optimistic. The choice of fabrication route strongly influences how well the three SABN functions are preserved during scale-up. Table 6 provides a qualitative ranking of the most frequently reported approaches against the criteria that matter for commercial packaging: mechanical integrity, barrier performance, retention of catalytic activity over time, control of nanoparticle migration, and overall industrial feasibility.

Overall, the central challenge is not the absence of scalable methods but the absence of validated, reproducible, and economically transparent workflows. Without pilot-scale demonstrations, robust batch-to-batch statistics, and full cost accounting, claims of industrial feasibility remain largely speculative. Compared to conventional ethylene-scavenging solutions such as KMnO_4_-based systems, SABNs offer multifunctionality (adsorption, catalysis, and in some cases responsiveness) and potential regenerability, which can translate into higher efficiency under optimized conditions. However, these advantages are offset by higher material and processing costs, higher formulation complexity, and very limited industrial validation. At the current stage, SABNs are better viewed as emerging complementary technologies rather than direct replacements for established systems.

### 6.2. Safety, Migration, and Regulatory Landscape

Nanoparticle migration remains the most persistent and consequential barrier to the adoption of SABN-enabled packaging. Multiple studies confirm that both ionic species and intact nanoparticles can leach into food matrices [89,90,91,92], yet the dominant testing paradigm of short-term exposure to simple food simulants fails to capture the complexity of real foods. Simulants such as 10% ethanol or 3% acetic acid routinely underestimate the release in lipid-rich, protein-rich, or high-sugar matrices [93,94], creating a systematic bias toward false reassurance. This systematic bias is plainly evident in Table 4, where the “Migration Assay” column remains almost entirely populated by N.R. (not reported), highlighting the fundamental disconnect between the materials innovation community and regulatory science requirements. Even proposed mitigation strategies such as encapsulation or immobilization onto biopolymers [95,96] lack long-term validation under realistic storage conditions, where temperature cycling, mechanical stress, and high humidity may compromise the structural integrity. Several material and environmental factors govern the extent of nanoparticle migration into food. Particle size is a first-order parameter: smaller particles diffuse more rapidly through polymer matrices, and particles below approximately 10 nm may pass through interfacial gaps or polymer-free volume more readily than larger aggregates [89,90]. Surface coating or encapsulation can either suppress or, in some cases, exacerbate release. A dense covalently grafted coating that anchors the particle to the polymer backbone can significantly reduce leaching, whereas loosely bound surfactants or weakly adsorbed capping agents may themselves desorb and facilitate particle detachment [95]. The composition of the food matrix also matters considerably. Acidic simulants such as 3% acetic acid can corrode certain metal and metal oxide nanoparticles, increasing ionic release, while lipid-rich matrices can extract hydrophobic particles or coatings that would remain stable in aqueous simulants [92]. The storage duration further amplifies these effects. Short-term migration tests lasting hours or a few days systematically underestimate the release compared to multi-week or multi-month exposures, particularly when temperature cycling and mechanical flexing of the package are considered [90]. Toxicological data remain similarly fragmented. While acute oral exposure studies suggest limited short-term risk, uncertainties surrounding chronic exposure, bioaccumulation, and genotoxicity persist [97,98]. The migration behavior depends strongly on the particle size, surface chemistry, and interactions with complex food matrices, yet current studies provide limited and sometimes inconsistent data, often generated under oversimplified conditions. The gap between short-term toxicological reassurance and long-term risk assessment is therefore one of the most critical weaknesses in the current evidence base. Without extended migration studies (weeks to months), matrix-specific toxicology, and harmonized detection limits capable of distinguishing ionic, molecular, and nanoparticulate species, regulatory approval will remain slow and public trust fragile. Regulatory frameworks for SABN-enabled packaging are fragmented and poorly aligned with the multifunctional nature of these materials. In the U.S., SABNs often fall outside conventional FCN/GRAS categories [72,95,99], while in the EU, strict migration limits exist for metals, but nanoparticle-specific guidance remains incomplete. Beyond the extent of migration, the potential for ingested nanoparticles to cause adverse health effects depends on their intrinsic toxicity and the exposure dose. Although a comprehensive toxicological review is beyond the scope of this manuscript, several key mechanisms have been identified for metal oxide nanoparticles commonly employed in SABNs. These include the generation of reactive oxygen species leading to oxidative stress, the induction of inflammatory responses, and, for certain materials, genotoxic effects (EFSA, 2011 [97]; Bumbudsanpharoke and Ko, 2015 [96]). The risk to consumers is a function of both hazard and exposure: even a highly toxic substance poses negligible risk if migration remains below the detection limit, while a relatively inert material may warrant concern if released in large quantities. Current risk assessment frameworks for food-contact nanomaterials, such as that proposed by the EFSA, therefore require the determination of a margin of exposure that compares the estimated daily intake from migration with a toxicological reference dose derived from in vitro and in vivo studies (EFSA, 2011 [97]; Cwiek-Ludwicka and Ludwicki, 2017 [98]). The critical challenge documented in this review is that migration data under realistic food-contact conditions are almost entirely absent (Table 3), which prevents even a preliminary risk characterization for any SABN system. Thus, the immediate priority is not an exhaustive toxicological profiling of every candidate material but the generation of the exposure data that risk assessment models require as input. The regulatory pathways for SABN-enabled packaging differ markedly between jurisdictions. In the United States, the FDA regulates food-contact substances under the Federal Food, Drug, and Cosmetic Act, generally requiring a Food Contact Notification (FCN) for new materials. Nanoscale substances are evaluated on a case-by-case basis, with the FDA recommending that manufacturers address the particle size, agglomeration, and potential migration in their submissions [95]. In the European Union, Regulation (EC) No. 1935/2004 sets an overall migration limit of 10 mg of total constituents per square decimeter of packaging surface, while Regulation (EU) No. 10/2011 establishes specific migration limits for authorized substances, including several metals relevant to SABNs such as titanium, zinc, and cerium. For engineered nanomaterials, the EFSA has published guidance requiring a detailed characterization of the particle size distribution, solubility, and migration behavior under intended use conditions [98]. Despite these frameworks, neither the FDA nor the EFSA has yet issued specific guidance for multifunctional active nanomaterials that combine adsorption, catalysis, and stimuli-responsive behavior. For active and intelligent packaging specifically, the European Union has established a separate regulatory framework under Regulation (EC) No. 450/2009, which stipulates that active components that deliberately release substances into the food or modify the headspace must be on a positive list and are subject to specific migration limits determined by toxicological evaluation. To date, no SABN component has been assessed under this dedicated framework. This regulatory gap, combined with the near-total absence of systematic migration data documented in this review, represents a critical bottleneck for commercialization. A deeper problem lies in the heterogeneity of experimental protocols across the literature: differences in light intensity, ROS-generating conditions, headspace spiking strategies, storage temperature, and migration assays [89,90] make cross-study comparison nearly impossible. This lack of harmonization undermines the reproducibility, complicates the exposure assessment, and slows regulatory review. For SABNs to progress toward commercialization, the field must converge on standardized food-relevant test protocols that (i) simulate realistic cold-chain and ambient supply-chain environments (including temperature cycling, humidity, and mechanical abrasion); (ii) incorporate long-duration migration experiments in representative food matrices; and (iii) specify analytical sensitivity and reporting standards for nanoparticle detection and speciation. Integration of in vitro digestion models, organoid systems, and long-term exposure assays into these frameworks will be essential to build regulatory confidence and to translate promising laboratory results into robust safety dossiers.

### 6.3. Environmental Life-Cycle Assessment (LCA)

While SABNs can reduce food waste by extending the shelf life, their own environmental footprint remains insufficiently characterized. Life-cycle considerations are increasingly important in evaluating advanced packaging technologies, because SABNs may simultaneously lower food waste-related emissions and introduce additional burdens associated with nanomaterial production, use, and end-of-life management. Preliminary LCAs suggest that low nanoparticle loadings in biodegradable matrices contribute modestly to CO_2_ emissions [99,100,101], yet these assessments often ignore toxicity pathways, nanoparticle persistence, and realistic disposal scenarios. Most existing studies focus narrowly on the carbon footprint and rely on simplified assumptions rather than cradle-to-grave analyses, creating a risk of overstating the environmental benefits. From a production standpoint, the synthesis of nanomaterials used in SABNs frequently relies on energy-intensive processes, organic solvents, and chemical precursors with non-negligible environmental footprints. As a result, the per-unit carbon footprint and resource demand of SABN production may exceed those of conventional packaging materials, particularly when high-purity reagents, multi-step syntheses, or stringent sterilization conditions are required. These upstream burdens are rarely captured in sufficient detail in current LCAs, which often treat nanoparticle inputs as marginal additives rather than as distinct process streams with their own environmental profiles. During the use phase, SABNs can contribute positively by reducing spoilage rates, extending the shelf life (typically on the order of 1–5 days), and thereby lowering food-waste-related emissions. For perishable and resource-intensive foods such as meat and dairy, the environmental benefit associated with avoided production, processing, and cold-chain transport can outweigh the additional material impacts of SABN-enabled packaging. However, many LCAs implicitly assume idealized performance gains and uniform shelf-life extension across products, perfect cold-chain management, and full consumer uptake conditions that may not hold in real supply chains. Without empirical product-specific data on spoilage reduction and waste avoidance, the magnitude of these use-phase benefits remains uncertain. End-of-life considerations introduce further complexity. Nanocomposite structures can be difficult to recycle due to incompatibilities with conventional sorting and reprocessing streams, and there is a potential for nanoparticle release during mechanical recycling, landfill leachate generation, or incineration. Current LCA models rarely incorporate these pathways or their ecotoxicological implications. Incorporation of SABNs into biodegradable or bio-based matrices (e.g., cellulose, PLA) offers a potential pathway to mitigate end-of-life issues, yet the long-term degradation behavior, nanoparticle release kinetics, and environmental fate of the resulting fragments remain insufficiently studied. A credible sustainability assessment must therefore integrate ecotoxicological endpoints, landfill and incineration scenarios, and realistic recycling limitations and must explicitly compare SABN systems with incumbent packaging solutions under equivalent functional units (e.g., kg of edible product delivered to consumer). Collectively, these gaps indicate that the environmental profile of SABN-enabled packaging is still provisional. Until LCA methodologies evolve to capture production-phase burdens, empirically grounded use-phase benefits, and end-of-life nanoparticle fate within a cradle-to-grave framework, claims of environmental superiority will remain tentative and vulnerable to regulatory or public scrutiny. Robust standardized LCAs aligned with emerging regulatory expectations will be essential to position SABNs as genuinely sustainable alternatives rather than merely technologically advanced solutions.

### 6.4. Public Perception and Labeling

Consumer acceptance is a decisive bottleneck for SABN commercialization. Surveys consistently show that the term “nano” evokes perceptions of artificiality or risk [102], meaning that even technically sound solutions may face market resistance. Transparent labeling and digital traceability tools such as QR-linked safety dossiers [103,104] can help, but communication must be carefully designed: poorly framed messages can amplify skepticism rather than alleviate it [105,106]. A further complication is the absence of harmonized labeling frameworks across jurisdictions, which creates inconsistency and confusion. Without coordinated terminology and messaging, consumer trust will remain fragile and adoption uneven across markets.

### 6.5. Emerging Directions

Next-generation SABNs aim to integrate sensing, antimicrobial activity, and self-cleaning functions into multifunctional platforms [107,108]. Sense-and-treat systems that detect spoilage markers and trigger antimicrobial release [42,109] represent a conceptual leap toward autonomous packaging. Integration with IoT-enabled monitoring [110] could further transform supply-chain management. Yet multifunctionality introduces new challenges: complex formulations reduce reproducibility, biogenic routes suffer from precursor variability, and controlled-release systems raise safety concerns. IoT integration adds cost, data-governance, and infrastructure burdens. The field’s future depends on balancing innovation with simplicity, demonstrating robust single-function reliability under real supply-chain conditions before layering additional capabilities. Notably, none of the systems catalogued in Table 4 integrate sensing, degradation, and IoT connectivity into a single validated platform, underscoring that these ambitious directions remain in the conceptual or early prototyping phase. Finally, it is worth noting that computational tools, particularly machine learning and molecular simulation, are beginning to influence the design of functional packaging materials. High-throughput screening of metal–organic frameworks, for example, has been used to identify candidates with optimal pore sizes and surface chemistries for selective gas adsorption [111]. Similarly, machine learning models trained on experimental datasets have shown promise in predicting photocatalytic activity and stability under varying environmental conditions. For SABNs, these approaches could, in principle, help navigate the large combinatorial space of scaffold–catalyst–trigger combinations, reducing the need for trial-and-error synthesis. However, the application of such tools to multifunctional active packaging remains in its infancy, and their predictive power is currently limited by the scarcity of systematic experimental data of the kind highlighted throughout this review.

### 6.6. Toward a Credible Roadmap and Future Outlook

Across all dimensions, technology readiness, safety, scalability, regulation, sustainability, and public trust, the central challenge for SABN-enabled packaging is not a lack of conceptual promise but a lack of validated, standardized, and food-relevant evidence. Smart adsorption-based nano-catalysts have demonstrated strong potential for next-generation active packaging, yet their transition from laboratory prototypes to industrially deployable technologies will depend on coordinated advances across materials science, food engineering, toxicology, environmental assessment, and regulatory science. Several strategic directions are likely to shape the next decade of research and commercialization:

- *Standardized cold-chain-relevant testing protocols.* Current evaluations of SABNs rely heavily on idealized laboratory conditions, controlled humidity, constant illumination, and simplified headspace compositions. Real supply chains, by contrast, involve low-light storage, fluctuating temperatures, high humidity, and complex food matrices. Future studies must adopt harmonized protocols that simulate realistic cold-chain and ambient environments, incorporate dynamic headspace analysis, and report performance across multiple batches, cultivars, and seasons. Without such standardization, cross-study comparison and industrial translation will remain limited.
- *Long-duration migration, toxicology, and matrix-specific risk assessment.* Migration remains the most critical barrier to regulatory approval. Short-term simulant tests underestimate the release in lipid-rich or protein-rich foods, and chronic exposure data are scarce. Next-generation SABNs will require multi-month migration studies, matrix-specific toxicological assessments, and validated analytical methods capable of distinguishing ionic, molecular, and nanoparticulate species. Integration of in vitro digestion models, organoid systems, and long-term exposure assays will be essential to build regulatory confidence.
- *Scalable, low-cost, and reproducible manufacturing routes.* Industrial adoption will require greener, low-temperature, and continuous manufacturing methods such as roll-to-roll deposition, reactive extrusion, or printing-based integration, combined with rigorous batch-to-batch quality control. Techno-economic analyses should incorporate nanoparticle stabilization, quality control, regulatory testing, and end-of-life handling to provide realistic cost models and should benchmark SABNs against incumbent technologies (e.g., KMnO_4_-based ethylene scavengers) on a cost-to-performance basis.
- *Integration with recyclable and bio-based packaging matrices*. As packaging regulations increasingly emphasize sustainability and circular economy principles, SABNs must be compatible with recyclable polymers, compostable biopolymers, and established waste-management infrastructures. Future research should explore immobilization strategies that prevent nanoparticle release while maintaining catalytic activity, and systematically evaluate how SABNs influence recyclability, biodegradation, and environmental fate using quantitative LCA.
- *Hybrid systems combining sensing, adsorption, and catalysis*. The next generation of active packaging is likely to merge SABNs with intelligent indicators, enabling the real-time monitoring of spoilage markers while simultaneously removing them. Such hybrid systems could provide adaptive control of headspace chemistry but will require careful design to avoid cross-interference between sensing and catalytic functions and to keep formulation complexity manageable. Integration with low-cost optical or electrochemical sensors and potentially with IoT-enabled monitoring represents a promising direction for high-value supply chains.
- *Consumer acceptance and transparent risk communication*. Even if SABNs meet regulatory requirements, consumer acceptance will depend on clear communication regarding their safety, migration, and environmental impact. Studies on consumer perception, labeling strategies, and risk-communication frameworks are currently lacking. Future commercialization efforts must incorporate social-science perspectives and coordinated terminology to ensure public trust and avoid fragmented messaging across jurisdictions. Among the systems surveyed, Ce-MOF/TiO_2_ composites (Hong et al., 2025) and TiO_2_@BMOF films (Riahi et al., 2025) stand out because they combine a well-characterized porous scaffold, a photocatalyst with documented ethylene degradation activity, and a responsiveness to light or humidity [29,67]. However, both are photocatalytic and therefore inactive in the dark; their further development for real supply chains will require either pairing with low-intensity visible-light sources or replacing the photocatalytic component with a nanozyme (e.g., CeO_2−x_ or Fe_3_O_4_) that functions without light. For dark cold-chain storage, the pH-responsive CeO2@nanocellulose pads represent a promising non-photocatalytic alternative, although they currently lack standardized migration data. No single system yet meets all three SABN criteria robustly under realistic cold-chain conditions while also being backed by pilot-scale cost analysis, but these named examples are, in our assessment, the most sensible starting points for the systematic progression outlined in the roadmap. Taken together, a credible roadmap for SABN commercialization must include the following: (i) long-duration matrix-specific migration and toxicology studies; (ii) harmonized test protocols reflecting real supply-chain stresses; (iii) full techno-economic and life-cycle models with sensitivity analyses; (iv) replicated pilot-scale trials across product categories; and (v) coordinated regulatory engagement and transparent consumer communication. Translating the conceptual promise of SABNs into commercial reality requires a phased approach. In the short term, the most urgent priority is to generate the missing evidence base: long-duration migration studies using real food matrices, sensory panels to detect off-flavors, and performance testing under standardized cold-chain conditions (dark, 4 °C, high humidity). Without these data, regulatory approval and industrial confidence will remain out of reach. Medium-term priorities should focus on scalable manufacturing: adapting laboratory syntheses to continuous processes such as roll-to-roll coating or reactive extrusion and rigorously assessing batch-to-batch reproducibility and cost at pilot scale. In the longer term, once safety, reliability, and economic feasibility have been demonstrated, research can turn to multifunctional platforms that integrate sensing, antimicrobial activity, or IoT connectivity. The most critical research gaps identified in this review are as follows: (i) the complete absence of systems that have reached evidence level L^5^ (standardized migration testing plus sensory evaluation); (ii) the lack of degradation product analysis confirming that partial oxidation intermediates are not harmful; (iii) the absence of cradle-to-grave life-cycle assessments that account for nanoparticle release during disposal; and (iv) the absence of validated techno-economic models comparing SABN costs to incumbent technologies on a per-unit-of-shelf-life-extension basis. By integrating standardized testing, robust safety validation, scalable manufacturing, and sustainability considerations, SABNs can evolve from compelling laboratory prototypes into credible industry-ready technologies capable of reducing food waste and improving global food-system resilience.

## 7. Conclusions and Future Perspectives

This review set out to examine whether SABNs can move beyond interesting chemistry and toward real food packaging practice. The short answer, based on the evidence gathered here, is not yet. But the path forward is becoming clearer. On the conceptual side, the SABN framework helps bring order to a fragmented field. By insisting that a genuine SABN must integrate an adsorptive scaffold, a catalytic center, and a stimuli-responsive trigger and by providing explicit inclusion and exclusion criteria (Table 1), we have drawn a boundary around what is otherwise a loose collection of nanocomposite materials. The bibliometric survey (Section 2.5) confirmed what many researchers in the area suspect: adsorption, photocatalysis, and responsive-packaging studies rarely speak to one another, and the integrated three-pillar perspective that defines SABNs is essentially absent from the existing review literature. On the experimental side, the comparative evidence summarized in Table 4 reveals a field still in its early demonstrative phase. The majority of systems operate at evidence levels L^3^ with real food under ambient light and room temperature, and only a handful have been tested in the cold-chain conditions (dark, 4 °C, high humidity) that matter commercially. No system in the surveyed literature has yet reached L^5^, a level that would require a standardized migration assay and a trained sensory panel alongside packaging performance data. This single observation explains why regulatory approval and industrial adoption remain distant goals. What needs to happen next is not mysterious. The community would benefit most from a deliberate, staged progression: (i) take the best-performing L^3^ and L^4^ candidates and generate long-duration migration and sensory data under realistic cold-chain and retail conditions (moving them to L^5^); (ii) accompany these studies with transparent reporting of the fabrication costs, energy consumption, and end-of-life considerations; (iii) establish a minimal set of harmonized testing conditions including darkness, refrigeration, and representative food matrices so that results from different laboratories can actually be compared; and (iv) delay adding extra functionalities (sensing, IoT, antimicrobial switches) until the core adsorption–catalysis–regeneration cycle has been demonstrated reliably and safely at scale. Equally important, the narrative around SABNs needs to be recalibrated. At present, these materials are best described as a promising conceptual platform with encouraging laboratory-scale data, not as commercially ready active-packaging solutions. Adjusting claims to match the available evidence will not weaken the case for SABNs; on the contrary, it will build the credibility that is needed for interdisciplinary collaboration, regulatory engagement, and eventual industrial uptake. In summary, SABNs represent a genuine conceptual advance from passive containment toward dynamic headspace management, but that advance has yet to be validated outside the laboratory. The translational gap documented in this review is large, yet it is also well-defined. To translate SABNs from laboratory prototypes into commercially viable packaging technologies, a staged progression is needed: (i) screening of candidate materials against the three-pillar inclusion criteria established in this review; (ii) performance validation under standardized cold-chain conditions (dark, 4 °C, high humidity) using representative food matrices; (iii) systematic migration assessment with recognized food simulants and long-duration exposure protocols; (iv) pilot-scale fabrication accompanied by transparent cost analysis and life-cycle assessment; and (v) compilation of a regulatory dossier addressing both food-contact safety and environmental fate. This sequence prioritizes the foundational evidence safety, reliability, and economic feasibility that must be in place before adding more advanced functionalities such as sensing or IoT connectivity. Finally, the development of SABN-enabled packaging should be viewed within the broader framework of the United Nations Sustainable Development Goals. Food loss and waste account for an estimated 8–10% of global greenhouse gas emissions, directly undermining SDG 13 targets for climate action [112]. Packaging technologies that extend shelf life and reduce postharvest losses can contribute meaningfully to SDG 12.3, which calls for halving per capita food waste by 2030. At the same time, the environmental footprint of the SABNs themselves must be carefully managed to avoid shifting the burden from food waste to plastic or nanoparticle pollution, a tension that aligns with the integrated vision of the 2030 Agenda. Closing it will require less emphasis on incremental material novelty and more on the systematic unglamorous work of testing, standardizing, and costing. If the research community makes that shift, SABNs stand a realistic chance of contributing meaningfully to food preservation and waste reduction. If it does not, they risk remaining an elegant laboratory concept with little practical footprint.

## Figures and Tables

**Table 1:** Inclusion and exclusion criteria for SABN classification.

Criterion	Included (SABN)	Excluded (Non-SABN)
Adsorptive scaffold	High-surface-area porous host intentionally designed for spoilage-marker capture (e.g., MOF, mesoporous silica, functionalized biopolymer matrix)	Passive polymer films without engineered porosity; conventional packaging materials
Catalytic center	Embedded catalytic domain that transforms adsorbed species (e.g., TiO_2_, ZnO, CeO_2_ nanozymes) with demonstrated activity under food-relevant conditions	Stand-alone photocatalysts without adsorptive scaffold; passive scavengers (e.g., KMnO_4_ sachets) that rely on stoichiometric oxidation without catalytic turnover *
Stimuli-responsive mechanism	Built-in trigger (light, pH, humidity, redox) that reversibly modulates activity or regenerates active sites in a switch-like manner	Systems whose activity is entirely independent of environmental cues; conventional adsorbents that lack any responsive functionality
Synergy requirement	Demonstrated or rationally designed coupling between adsorption and catalysis (e.g., scaffold concentrates analyte near catalytic site; catalysis regenerates adsorption capacity)	Independent adsorbent + catalyst mixtures without intentional integration; multilayer structures where adsorption and catalysis occur in physically separated layers without cooperative interaction
Evidence base	Studies reporting at minimum headspace analysis under controlled conditions	Purely computational predictions without experimental validation; systems tested only in non-food environments (e.g., industrial wastewater) without food packaging contextualization

* This excludes antimicrobial agents that function primarily through ion release (e.g., Ag^+^) or stoichiometric oxidation, as they lack sustained catalytic turnover.

**Table 2:** Core design dimensions and functional roles of SABNs.

Design Dimension	Typical Material Platforms	Primary Function in SABNs	Key Design Considerations	References
Adsorptive Scaffold	Mesoporous silica, hierarchical TiO_2_, graphene oxide, MOFs	Capture of ethylene, VOCs, and mycotoxins via physical/chemical adsorption	Surface area (>200 m^2^/g), pore accessibility, selectivity	[33]
Catalytic Center	TiO_2_, ZnO, CeO_2_, Fe_3_O_4_, metal or CQD hybrids	In situ degradation of adsorbed species into benign products	Band structure, ROS generation, catalyst stability	[30,34]
Stimuli Responsiveness	Light-activated oxides, humidity-responsive polymers, redox-active ceria	On-demand activation, regeneration, and adaptive response	Trigger sensitivity, reversibility, environmental robustness	[12]

**Table 3:** Summary of food categories, dominant spoilage markers, commonly studied SABN materials, and typical testing conditions. Details and primary references for each material system are provided in the corresponding subsections of Section 5.

Food Category	Main Spoilage Markers	Commonly Studied SABN Materials	Typical Testing Conditions
Fruits and vegetables	Ethylene, aldehydes	TiO_2_-based films, MOF composites, zeolite–TiO_2_ hybrids, CNF/TNT-CuO films	20–25 °C, ambient or fluorescent light, moderate RH
Meat and seafood	Biogenic amines (TVB-N), sulfides, microbial metabolites	CeO_2_ nanozymes, pH-responsive pads, chitosan-based films, Ag/TiO_2_ composites	4 °C, dark or low light, high humidity, intact tissue
Dairy products	VOCs, lipid oxidation products, microbial off-odors	Ag/graphene/TiO_2_-PLA films, TiO_2_/essential oil bio-nanocomposites	4 °C, dark, direct cheese contact
Bakery products	Staling-related VOCs, aldehydes, moisture	Chitosan/TiO_2_ films, carbon dot-functionalized biopolymers	20–25 °C, ambient light, moderate RH
Multilayer and sachets	Ethylene, VOCs (broad spectrum)	Hybrid laminates, active sachets, indicator-integrated systems	Variable; often ambient conditions, limited cold-chain data

**Table 5:** Qualitative comparison of conventional petroleum-based films with SABN-enabled systems based on conventional and bio-based polymer matrices. The ratings are qualitative summaries of the evidence reviewed in Section 3, Section 4 and Section 5 and the scalability discussion in Section 6.1; specific references for individual material systems are compiled in Table 4.

Criterion	Conventional Petroleum-Based Films	SABNs on Conventional Polymers	SABNs on Bio-Based Polymers
Barrier properties (O_2_, H_2_O)	+++ *	++ to +++	+ to ++
Active headspace control	−	++	++
Shelf-life extension	−	+ to ++	+ to ++
Scalability	+++	+	+
Relative cost	Low	Moderate to high	High
End-of-life/environmental impact	High (persistent waste)	Moderate (nanoparticle release concern)	Lower (biodegradable matrix, nanoparticle fate still uncertain)

* Symbols: +++ = excellent/high, ++ = good/moderate, + = limited/low, − = absent/negligible. The qualitative ratings are based on trends observed across the studies reviewed in Section 3, Section 4 and Section 5 and the commercial benchmarks discussed in Section 6. Detailed performance data and references for each SABN system are provided in the comparative evidence in Table 4.

**Table 6:** Qualitative comparison of common fabrication approaches for integrating SABNs into packaging materials *.

Approach	Mechanical Performance	Barrier Properties	Stability (Activity Retention)	Release Control (Migration)	Industrial Feasibility
Direct blending (melt/solvent)	++	+	+	+	+++
In situ synthesis	+++	++	+++	++	+
Electrospinning	++	+++	+++	++	++
Layer-by-layer assembly	+	+++	++	+++	+
Coating / lamination	++	++	++	+	+++

* Symbols: +++ = high, ++ = moderate, + = low. Rankings are based on qualitative trends observed across the studies reviewed in Section 3, Section 4 and Section 5.

## Data Availability

No new data were created or analyzed in this study. Data sharing is not applicable to this article.
